# *Xenopus frizzled-4S*, a splicing variant of *Xfz4 *is a context-dependent activator and inhibitor of Wnt/β-catenin signaling

**DOI:** 10.1186/1478-811X-3-12

**Published:** 2005-10-19

**Authors:** Rajeeb Kumar Swain, Masaru Katoh, Araceli Medina, Herbert Steinbeisser

**Affiliations:** 1 Institute of Human Genetics, University of Heidelberg, Im Neuenheimer Feld 366, 69120 Heidelberg, Germany; 2 Deptartment of Cell Biology, Max Planck Institute for Developmental Biology, Spemann Str. 35, 72076 Tübingen, Germany; 3 Genetics and Cell Biology Section, Genetics Division, National Cancer Center Research Institute, Tsukiji 5-chome, Chuo-ku, Tokyo, 104-0045, Japan

## Abstract

**Background:**

Secreted Frizzled related proteins (SFRPs) are extracellular regulators of Wnt signaling. These proteins contain an N-terminal cysteine rich domain (CRD) highly similar to the CRDs of the Frizzled family of seven-transmembrane proteins that act as Wnt receptors. SFRPs can bind to Wnts and prevent their interaction with the Frizzled receptor. Recently it has been reported that a splice variant of human Frizzled-4 (FZD4S) lacking the transmembrane and the cytoplasmic domains of Frizzled-4 can activate rather than inhibit Wnt-8 activity in *Xenopus *embryos. This indicates that secreted CRD containing proteins such as Frizzled ecto-domains and SFRPs may not always act as Wnt inhibitors. It is not known how FZD4S can activate Wnt/β-catenin signaling and what biological role this molecule plays *in vivo*.

**Results:**

Here we report that the *Xenopus frizzled-4 *is alternatively spliced to give rise to a putative secreted protein that lacks the seven-transmembrane and the cytoplasmic domains. We performed functional experiments in *Xenopus *embryos to investigate how this novel splicing variant, Xfz4S, can modulate the Wnt/β-catenin pathway. We show that Xfz4S as well as the extracellular domain of Xfz8 (ECD8) can act as both activators and inhibitors of Wnt/β-catenin signaling dependent on the Wnt ligand presented. The positive regulation of Wnt/β-catenin signaling by the extracellular domains of Frizzled receptors is mediated by the members of low density lipoprotein receptor-related protein (LRP-5/6) that act as Wnt coreceptors.

**Conclusion:**

This work provides evidence that the secreted extracellular domains of Frizzled receptors may act as both inhibitors and activators of Wnt signaling dependent on the Wnt ligand presented.

## Background

Wnts are secreted glycoproteins that control an array of signaling processes in embryos and adult tissues [1-4]. These proteins act through the members of the Frizzled family of seven-transmembrane receptors [5,6]. Wnt and Frizzled interaction leads to the stabilization of cytoplasmic β-catenin, its nuclear translocation and subsequent transcriptional activation of Wnt/β-catenin target genes [1,7]. Two members of low-density lipoprotein receptor-related protein, LRP-5 and -6, act as coreceptors in the Wnt/β-catenin signaling [8-10]. These transmembrane proteins can interact with Wnts and form a ternary complex with Frizzled receptors [9]. This leads to the binding of axin to the cytoplasmic domain of LRP and its recruitment to the membrane [11]. Axin is a scaffolding protein necessary in the cytoplasm for assembly of the protein complex that phosphorylates β-catenin and promotes its degradation by ubiquitin proteasome dependent pathway [12,13]. Recruitment of axin to the membrane by LRP leads to the reduced phosphorylation of β-catenin and subsequent activation of Wnt/β-catenin pathway. In the extracellular space, various secreted molecules negatively regulate Wnt/β-catenin signaling [14]. Prominent among them are the members of the secreted Frizzled related protein family (SFRP) that inhibit Wnt/β-catenin signaling primarily by binding to the Wnts and preventing Wnt/Frizzled interaction. Dickkopf family of extracellular proteins can bind to the Frizzled coreceptor LRP-5/6 and inhibit Wnt/β-catenin signaling [15,16]. SFRPs contain a cystein rich domain (CRD) that is also found in the Frizzled receptors [14]. The CRD of Frizzleds and SFRPs is required for their interaction with Wnts [5,14]. In this paper, we present evidence that *Xenopus frizzled-4 *is alternatively spliced to generate a putative secreted protein (Xfz4S), containing a part of the extracellular domain but lacking the seven-transmembrane and cytoplasmic domains. Xfz4S can activate or inhibit the Wnt/β-catenin signaling dependent on the Wnt ligand presented. We show that the extracellular domain of *Xenopus *frizzled-8 (ECD8) not only inhibits Wnt signaling induced by a variety of Wnt ligands, but can also act synergistically with Wnt-5a in inducing Wnt/β-catenin signaling. We further show that the activation of Wnt/β-catenin pathway by the extracellular domains of Frizzled receptors is dependent on LRP.

## Results

### *Xenopus frizzled- 4 *is alternatively spliced

**Figure 1:**
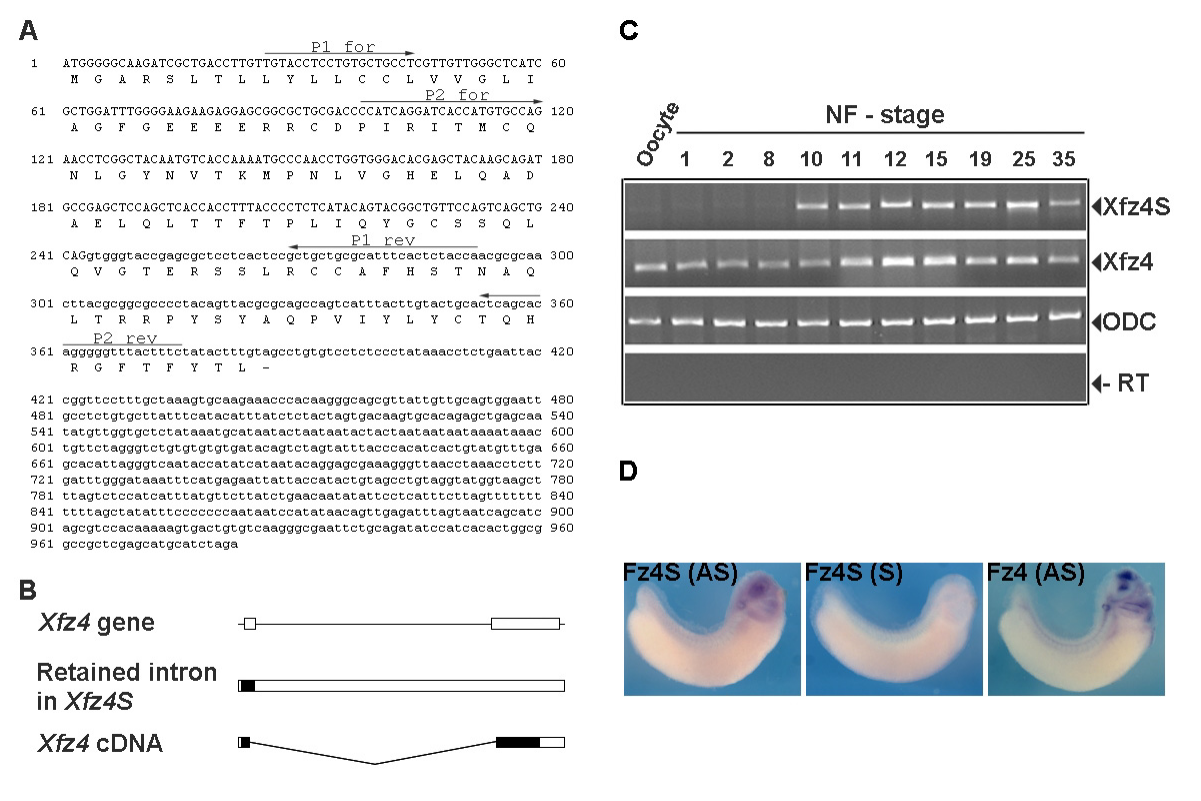
Molecular structure and expression pattern of *Xfz4S*. (A) Nucleotide and amino acid sequences of *Xfz4S*. The nucleotide sequence of Exon1 of *Xfz4 *is in capital and that of Intron1 is in small letters. Two sets of primers (P1F/P1R and P2F/P2R) were used for detection of *Xfz4S *by RT-PCR. (B) Schematic diagram showing the structure of *Xfz4 *gene containing two exons (boxes) and one intron. The splicing variants, *Xfz4S *retaining the intron and the *Xfz4 *are shown. The coding regions of these splicing variants are indicated by closed boxes and the UTRs by open boxes. (C) Developmental RT-PCR of *Xenopus *embryos with indicated Nieuwkoop and Faber (NF) stages. Xfz4S mRNA is first detected after mid blastula transition and the expression persist into tadpole stages. *Xfz4 *mRNA is maternally supplied and is expressed in all stages of development studied. ODC is a loading control. (D) Spatial expression pattern of Xfz4 and Xfz4S in tailbud stage embryos (stage 34). The embryos were hybridized with digoxigenin labelled RNA probes for antisense Xfz4 (Xfz4-AS), antisense Xfz4-intronI (Xfz4S-AS) or sense probe for Xfz4-intronI (Xfz4S-S).

It has been reported that the human Frizzled 4 (FZD4) is alternatively spliced to give rise to a transcript (*FZD4S*) in which the intron-1 of FZD4 is retained [17]. In order to investigate if *Xenopus frizzled*-4 (*Xfz4*) could also be alternatively spliced, the genomic organization of this gene was studied. The *Frizzled-4 *gene in humans, *Xenopus laevis *and *Xenopus tropicalis* (), consists of 2 exons and one intron. In *X. laevis*, the intron has a length of 6 kb (Fig. 1A, 1B and unpublished observations of MK). Thus, the exon-intron structure of *Frizzled-4 *genes is conserved in human, mouse, *X. laevis *and *X. tropicalis*. The conserved exon-intron structure of the vertebrate *Frizzled-4 *genes prompted us to investigate if *Xenopus frizzled-4 *is alternatively spliced like its human ortholog. This putative splicing variant of Xfz4 (Xfz4S) in which the intron is retained should generate a protein of 128 amino acids. The first 81 amino acids of which are identical to the seven-transmembrane type Xfz4 and the other 47 amino acids are unique to Xfz4S (Fig. 1A and 1B). To determine if Xfz4S is expressed during the *Xenopus *development, RT-PCR was performed using PCR primers which recognize only the splicing variant of *Xfz4 *(Fig. 1A). The forward primers were selected from the exon-1 and the reverse primers were selected from intron-1. All the RNA samples were treated with DNaseI to eliminate any genomic DNA from the RNA preparation. The results show that *Xfz4 *is alternatively spliced and that the intron is retained in this splicing variant (Fig. 1C and 1B). Developmental RT-PCR showed that *Xfz4S *is expressed only after mid blastula transition (MBT) and expression persists during all stages of development studied (Fig. 1C). In contrast, *Xfz4 *message is maternally supplied and is present during all examined stages of *Xenopus *development (Fig. 1C and Ref. [18]). In order to study the spatial distribution of *Xfz4S *mRNA, a part of the intron1 of *Xfz4 *(*Xfz4-intron1*) was used as an *in situ *probe as this will specifically recognize the *Xfz4S *transcripts and not that of *Xfz4*. The *in situ *pattern of *Xfz4S *shows that this mRNA is ubiquitously expressed after MBT (data not shown). At the tail bud stage (stage 34), the strongest staining was observed in the head (Fig. 1D). The expression pattern of Xfz4S is not identical with Xfz4 but there is overlapping expression in the eye.

### Xfz4S acts synergistically with a specific group of Wnt ligands

**Figure 2:**
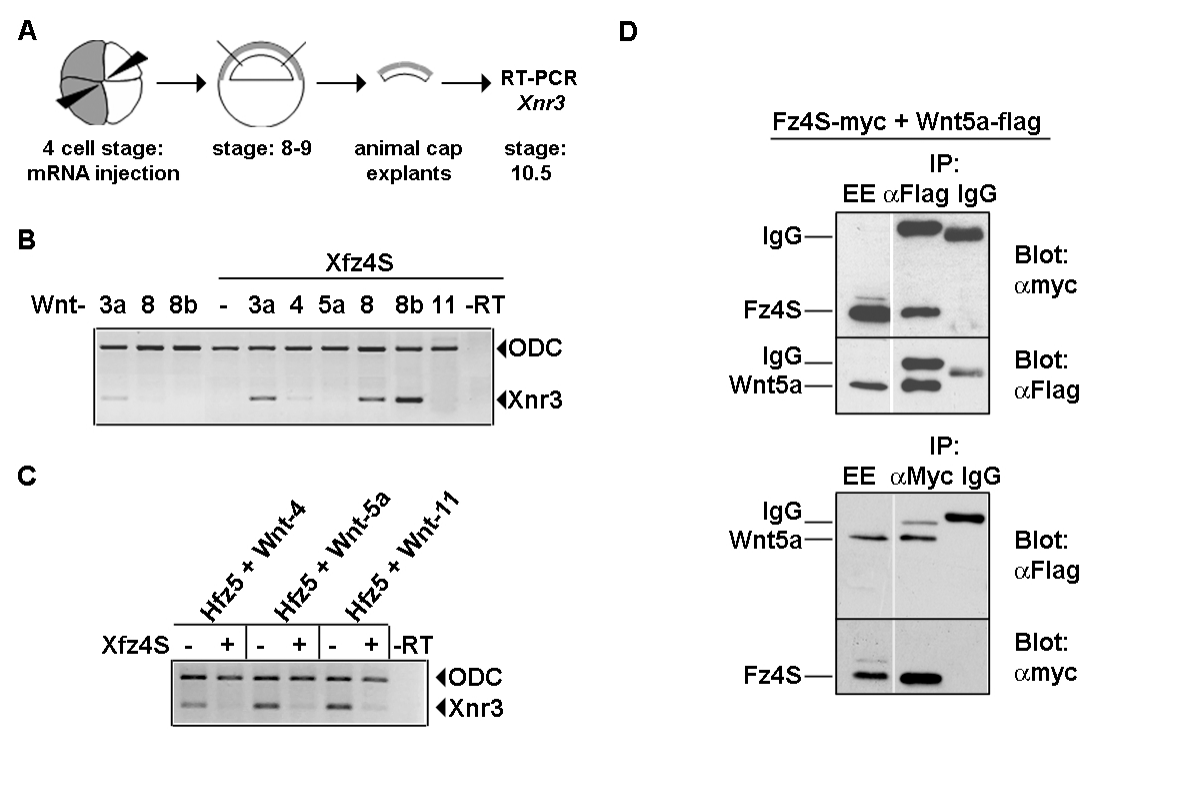
Xfz4S acts synergistically with canonical Wnt ligands in activating the Wnt/β-catenin target gene *Xnr3 *and inhibits non-canonical Wnt ligands. (A) Experimental scheme. Embryos were injected at 4 cell stage into the animal blastomeres with synthetic mRNA for *Xwnt-3a *(0.5 pg/embryo), *Xwnt-4 *(150 pg/embryo), *Xwnt-5a *(50 pg/embryo), *Xwnt-8 *(0.5 pg/embryo), *Xwnt-8b *(15 pg/embryo) or *Xwnt-11 *(50 pg/embryo), either alone or in combination with 500 pg *Xfz4S*. Animal caps were dissected out at stage 8 – 9, grown until stage 10.5 at which expression of *Xnr3 *was analyzed by RT-PCR. (B) *Xwnt-3a*, -*8*, -*8b *induce *Xnr3 *expression only in combination with *Xfz4S*. *Xwnt-5a*, -*4 *or -*11 *do not synergize with *Xfz4S *in inducing *Xnr3*. (C) Xfz4S inhibits *Xnr3 *expression induced by coinjection of Hfz5 and non-canonical Wnt-5a class ligands. Wnt-5a class ligands such as *Wnt-4 *(150 pg/embryo), -*5a *(50 pg/embryo) or -*11 *(50 pg/embryo) when injected in combination with 250 pg *Hfz5*, *Xnr3 *expression was induced. Induction of *Xnr3 *expression by these Wnt ligands in combination with *Hfz5 *was inhibited by coinjection of 500 pg *Xfz4S *mRNA. (D) Coimmunoprecipitation of Xfz4S and Wnt-5a. Myc-tagged Xfz4S (500 pg/embryo) and flag-tagged Wnt-5a (500 pg/embryo) were injected into *Xenopus *embryos at 2–4 cell stage. Myc-tagged Xfz4S coimmunoprecipitates with flag-tagged Wnt-5a indicating that they interact. A part of the embryo extract was incubated with mouse IgG, which serves as a control against non-specific binding of proteins. Total embryo extract (EE) shows the expression of Xfz4S-myc and Wnt5a-flag constructs.

Next, we tested the ability of Xfz4S to modulate the Wnt/β-catenin signaling. It has been shown, that FZD4S, a splice variant of human Frizzled-4 can enhance the activity of Wnt-8 in inducing secondary body axis when injected into the ventral marginal zone of *Xenopus *embryos [17]. This prompted us to ask if Xfz4S has similar activity in positively regulating Wnt/β-catenin signaling. Xfz4S, which is a putative secreted protein, may also inhibit Wnt signaling by sequestering the Wnts in the extracellular space. To test these possibilities, synthetic mRNA encoding *Xfz4S *was injected either alone or in combination with *Wnt *ligands into the animal blastomeres at 4-cell stage and the activation of Wnt/β-catenin target gene *Xnr3 *was monitored by RT-PCR at stage 10.5 (Fig. 2A). mRNAs for Wnt-1 type ligands (*Wnt-3a*, -*8 *or -*8b*) which can induce Wnt/β-catenin signaling were titrated to such low doses that they did not induce *Xnr3 *expression. When these Wnts were coinjected with *Xfz4S*, *Xnr3 *expression was induced (Fig. 2B). This suggests that Xfz4S acts synergistically with Wnt-3a, -8 and -8b in activating the Wnt/β-catenin pathway.

### Xfz4S can inhibit the Wnt activity

In our experiments, Xfz4S was not able to synergize with non-canonical Wnts such as Wnt-4, -5a or -11 in activating Wnt/β-catenin pathway (Fig. 2B). This could be due to the inability of these ligands to interact with Xfz4S. To test the interaction of Xfz4S and non-canonical Wnts we took advantage of the fact that the non-canonical Wnts that do not activate Wnt/β-catenin pathway when expressed alone, can do so in combination with Hfz5 [19]. We injected *Wnt-4*, -*5a *or -*11 *in combinations with *Hfz5 *into the animal blastomeres at 4-cell stage and monitored the expression of Wnt/β-catenin target gene *Xnr3 *by RT-PCR at stage 10.5. As expected, *Xnr3 *expression was induced in these animal caps. Coinjection of *Xfz4S *inhibited the activation *Xnr3 *by *Hfz5 *and *Wnt-4*, -*5a *or -*11 *(Fig. 2C). This suggests that Xfz4S can interact with non-canonical Wnts and can act as an inhibitor of the Wnt/β-catenin signaling.

Consistent with the functional interaction between Xfz4S and Wnt ligands in modulating the Wnt/β-catenin signaling, we found that myc-tagged Xfz4S coimmunoprecipitates with flag-tagged Wnt-5a (Fig. 2D). A flag-epitope tagged Xfz4S also coimmunoprecipitated with myc-tagged Wnt-11 (data not shown) indicating that Xfz4S forms a complex with these Wnt ligands.

### The extracellular domain of Xfz8 can activate Wnt/β-catenin pathway

**Figure 3:**
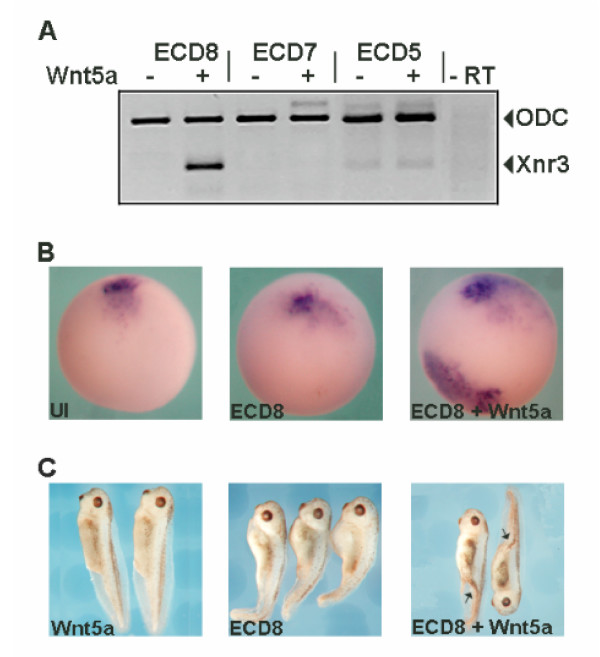
Extracellular domain of Xfz8 (ECD8) can activate Wnt/β-catenin pathway in combination with Xwnt-5a. (A) 500 pg/embryo *ECD8 *mRNA when injected in conjunction with *Xwnt-5a *(50 pg/embryo), *Xnr3 *expression was induced in animal cap tissues. Extracellular domains of *Xfz7 *(*ECD7*, 300 pg/embryo) or *Hfz5 *(*ECD5*, 500 pg/embryo) did not synergize with *Xwnt-5a *in inducing *Xnr3 *expression. (B) *ECD8 *(200 pg/embryo) and *Wnt-5a *(50 pg/embryo) when injected into the ventral marginal zone, *Xnr3 *expression was induced. (C) At later stages these embryos developed partial secondary axis without head structures. No secondary axes were observed when *ECD8 *or *Wnt-5a *was injected alone.

Based on our observation that Xfz4S, which resembles the extracellular domain of a Frizzled receptor can act as a positive regulator of Wnt signaling, we tested if the extracellular domains of other Frizzled receptors could exhibit the same function. Extracellular domains of Xfz7, Xfz8 and Hfz5 were tested in combination with both canonical and non-canonical Wnt ligands for their ability to modulate Wnt/β-catenin signaling. The Frizzled ecto-domains were injected into animal blastomeres of *Xenopus *embryos, either alone or in combination with *Wnt-3a*, -*4*, -*5a*, -*8*, -*8b *or -*11 *mRNA. Neither low dose of canonical Wnts alone, nor in combination with *ECD-7/-8/-5 *were able to induce *Xnr3 *expression in explanted animal cap tissues. Similar results were obtained when these ecto-domains of Frizzled receptors were injected in combination with *Wnt-4 *or *Wnt-11*. When *ECD8 *was coexpressed with *Wnt-5a*, however, *Xnr3 *expression was induced (Fig. 3A). Coexpression of *ECD8 *and *Wnt-5a *in the ventral marginal zone of the *Xenopus *embryos resulted in ectopic expression of *Xnr3 *(Fig. 3B). In later stages, such embryos developed incomplete secondary body axes without head structures (Fig. 3C). The activation of the Wnt/β-catenin target gene *Xnr3 *and the induction of secondary axes were not observed after injection of either *ECD8 *or *Wnt-5a *alone. It has been reported that expression of high amounts of *ECD8 *mRNA in the ventral marginal zone can induce secondary body axis including head structures [20]. The induction of such type of axis by ECD8 is achieved by inhibition of Wnt and BMP signaling and is not accompanied by induction of *Xnr3 *expression. In contrast to the induction of secondary axis structures by high doses of ECD8, the secondary axis induced by low doses of ECD8 and Wnt-5a was accompanied by induction of Wnt/β-catenin target gene *Xnr3 *(Fig. 3B). This provides further evidence that ECD8 and Wnt-5a act cooperatively in activating the Wnt/β-catening pathway.

### Activation of Wnt/β-catenin pathway by Xfz4S and ECD8 is mediated by LRP

**Figure 4:**
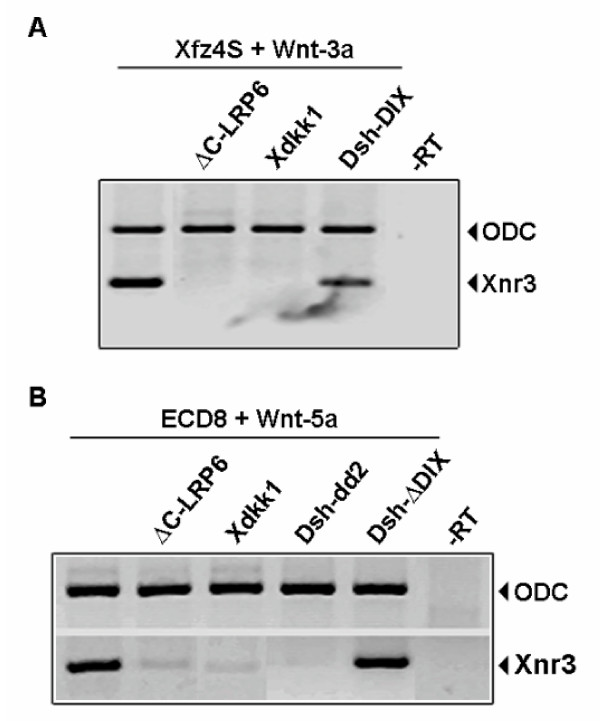
Activation of Wnt/β-catenin pathway by Xfz4S and ECD8 is LRP dependent. (A) *Xfz4S *(500 pg/embryo) and *Xwnt-3a *(0.5 pg/embryo) when coinjected into the animal caps, expression of *Xnr3 *was induced. This activation of *Xnr3 *was blocked by coinjection of 300 pg *Xdkk1 *or 1 ng *ΔC-LRP6 *and was greatly reduced by 250 pg *Xdsh-DIX*. (B) Induction of *Xnr3 *expression by injection of *ECD8 *(500 pg/embryo) and *Xwnt-5a *(50 pg/embryo) was blocked by coinjection of 300 pg *Xdkk1*, 1 ng *ΔC-LRP6 *or by 250 pg *Dsh-dd2*. Coinjection of a dishevelled mutant lacking the DIX domain (*Dsh-ΔDIX *250 pg/embryo) had no effect on ECD8 plus Wnt-5a induced activation of *Xnr3*.

We next asked how Frizzled ecto-domains that lack the transmembrane and the cytoplasmic domains could positively regulate Wnt/β-catenin signaling. Our working hypothesis is that the activation of Wnt/β-catenin signaling by the Frizzled ecto-domain is mediated by the Wnt coreceptor LRP5/6. We postulate that the Wnt-1/Xfz4S and Wnt5a/ECD8 complexes interact with the coreceptor LRP5/6 and activate the Wnt/β-catenin signaling in LRP5/6 and axin dependent manner. To test this hypothesis, we interfered with LRP mediated signaling in several ways. Xdkk1, a Wnt inhibitor, can bind directly to LRP5/6 and prevent LRP-Wnt-Frizzled ternary complex formation [15,16]. A LRP mutant lacking the cytoplasmic carboxy terminal domain (△CLRP) can not interact with axin but can sequester the Wnt-Frizzled complexes and prevent their interaction with endogenous LRP5/6 [9,11]. We also interfered with LRP-axin interaction by overexpression of the DIX domain of Xdsh. The DIX domain of Axin is required for its interaction with both disheveled and LRP [11,21]. Hence, overexpression of the Xdsh-DIX will sequester axin in the cytoplasm and will prevent its interaction with LRP. Low doses of *Wnt-3a *mRNA were injected into the animal caps in combination with *Xfz4S *to induce Wnt signaling as monitored by the activation of Wnt/β-catenin target gene *Xnr3*. The activation of *Xnr3 *expression was blocked by coexpression of Xdkk1 or ΔCLRP6 and was substantially reduced by coexpression of Xdsh-DIX (Fig. 4A). The Xdsh-dd1 mutant containing the DIX domain completely blocked Wnt signaling induced by *Wnt-3a *and *Xfz4S *(data not shown).

We employed the same strategy in animal cap assays to investigate if the activation of Wnt/β-catenin pathway by ECD8 and Wnt-5a is LRP dependent. When ECD8 was expressed together with *Wnt-5a *in animal caps, *Xnr3 *expression was induced. The activation of *Xnr3 *expression by ECD8 plus Wnt-5a was blocked by coinjection of either *Xdkk1 *or Δ*CLRP6*. A mutant Xdsh molecule lacking the carboxy-terminus DEP domain but containing the DIX domain (Xdsh-dd2) was also able to block *Xnr3 *expression induced by *ECD8 *and *Wnt-5a*. The Xdsh-dd1 and the Xdsh-DIX mutants both blocked Wnt-5a/ECD8 induced Wnt siganling (data not shown). Consistent with our argument, Xdsh mutant lacking the DIX domain (Xdsh-ΔDIX) was not able to interfere with *ECD8 *and *Wnt-5a *induced activation of *Xnr3 *(Fig. 4B). These results suggest that Wnt-3a/Xfz4S and Wnt-5a/ECD8 complexes can interact with LRP and activate the Wnt/β-catenin pathway in LRP-axin dependent manner.

## Discussion

### Regulation of Wnt signaling by a novel splice variant of Frizzled-4

In this study we report that *Xenopus frizzled-4 *is alternatively spliced to give rise to a transcript (*Xfz4S*) that is predicted to generate a secreted protein lacking the transmembrane and cytoplasmic domains. *Xfz4S *mRNA is expressed as a zygotic transcript and is present during all stages of *Xenopus *development (Fig. 1C).

**Figure 5:**
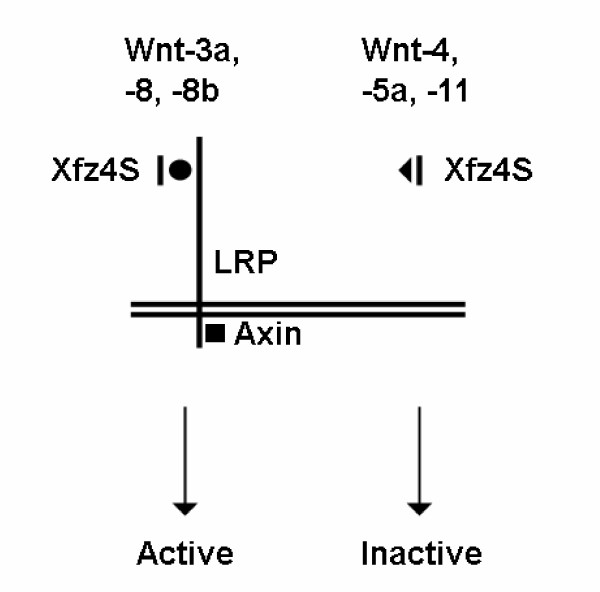
Model for the modulation of Wnt/β-catenin pathway by extracellular domains of Frizzled receptors. We propose that a complex formed between Xfz4S and Wnt-3a/-8/-8b could be recognized by LRP and Wnt signaling could be activated in LRP dependent manner. Complexes between Xfz4S and Wnt-4/-5a/-11 would not be recognized by LRP and Xfz4S and Wnt/β-catenin signaling would not be activated by these Wnts.

Xfz4S when overexpressed alone in *Xenopus *embryos, does not activate Wnt/β-catenin signaling. When coexpressed with Wnt-1 type ligands such as Wnt-3, -8 and -8b, it acts synergistically with these ligands in activating Wnt/β-catenin target gene *Xnr3 *(Fig. 2B). This is in agreement with the observation that human FZD4S acts synergistically with Wnt-8 in activation Wnt/β-catenin signaling [17]. Our results show that the ability of Xfz4S to synergize with Wnt ligands in activating Wnt/β-catenin signaling is dependent on Wnt coreceptor LRP (Fig. 4A). When Xfz4S was coexpressed with the non-canonical Wnt ligands such as Wnt-4, -5a and -11, it inhibited the ability of these ligands to activate Wnt/β-catenin signaling in conjunction with the receptor Hfz5 (Fig. 2C). This shows that Xfz4S can interact with both the canonical Wnt-1 and non-canonical Wnt-5a class ligands, but has opposite effects on Wnt/β-catenin signaling. This can be explained by postulating that only the complex between Xfz4S and the Wnt-1 type ligands is recognized by LRP and the Wnt/β-catenin pathway is activated in a LRP dependent manner whereas Wnt-5a/Xfz4S complex is not recognized by LRP. In this situation, Xfz4S will act as a negative regulator of Wnt/β-catenin signaling (Fig. 5).

### Dual role of Frizzled ecto-domains: activation and repression

It has been shown that the secreted Frizzled related proteins (SFRPs) and Frizzled ecto-domains act by binding to Wnts and sequestering them in the extracellular space. Contrary to this view, we show that Xfz4S that resembles the ecto-domain of Frizzled receptor can act as a positive regulator of Wnt signaling with a specific group of Wnt ligands (Fig. 2B). We also show that the ecto-domain of Xfz8 can act synergistically with Wnt-5a in activating Wnt/β-catenin signaling in a LRP dependent manner (Fig. 3 and 4B). This seems to contradict a report in which expression of *Xnr3 *induced by Frizzled-8 and Wnt-5a was inhibited by ECD8 [20]. It is plausible however, that full length Frizzled-8 is more potent than ECD8 in activating *Xnr3 *in combination with Wnt-5a. This interpretation is supported by our finding that coinjection of ECD8 and Wnt-5a only induced partial secondary body axes, whereas coexpression of full length Frizzled-8 and Wnt-5a induced complete secondary axes in *Xenopus *embryos (Fig. 3C and data not shown). In the presence of Frizzled-8, ECD8 and Wnt-5a, *Xnr-3 *expression should be reduced compared to the combination Frizzled-8 and Wnt-5a.

Our results suggest that Frizzled ecto-domains may not exclusively act as inhibitors of Wnt signaling. Similar observation has been made in case of Drosophila Frizzled-2 [Dfz2; [22]]. A mutant Dfz2 lacking the carboxy-terminal cytoplasmic domain (Dfz2△C) can synergize with Wingless (Wg) in transmitting Wnt/β-catenin signaling. Although Dfz2△C retains the seven-transmembrane domains, which may play a role in this signaling, our results would suggest that a Dfz2 mutant containing only the ecto-domain may be sufficient to synergize with Wg in activating this pathway. It has also been reported that SFRP2 can antagonize SFRP1 function during metanephric kidney development. In this process SFRP1 inhibits Wnt-4 signaling whereas SFRP2 promotes it [23]. These observations suggest that SFRPs may activate or inhibit Wnt signaling in a context dependent manner. Such dual activities have also been described for proteins of the Dkk family. Dkk2 can activate Wnt/β-catenin signaling and it synergizes with Frizzled receptors as well as with LRP6 in activating this pathway; whereas Dkk1 is an inhibitor of Wnt signaling [24,25]. These data indicate that the activity of extracellular factors which modulate Wnt signaling activity is dependent on the type of Wnt ligand and the cellular context. The biological significance of such dual activity, however, is poorly understood and will be a priority for future work. Although it is assumed that the non-canonical Wnts such as Wnt-5a and Wnt-11 function in β-catenin independent manner, it is not clear, if these Wnts may have functions mediated by β-catenin *in vivo*. Overexpression of Wnt-5a has been shown to correlate with abnormal nuclear localization of β-catenin protein in phyllodes tumor and ectopic Wnt-11 can rescue axis structures in UV ventralized *Xenopus *embryos by activation of the Wnt/β-catenin pathway [26,27]. Maternal Wnt-11 has been shown to activate Wnt/β-catenin signaling required for axis specification in *Xenopus *whereas zygotic Wnt-11 regulates non-canonical Wnt signaling, which coordinates gastrulation movements later in development [28-30]. This indicates that the activities of Wnt ligands in activating the canonical or non-canonical Wnt signaling may be regulated by extracellular cofactors. Supporting this hypothesis, Exostosin, an enzyme necessary for heparan sulfate proteoglycans (HSPGs) biosynthesis and EGF-CFC protein FRL1 have been shown to modulate Wnt-11 activity [28]. We postulate that secreted Frizzled related proteins and Frizzled ecto-domains may regulate the activation of distinct downstream signaling pathways triggered by Wnts.

## Conclusion

We conclude that the ecto-domains of Frizzled receptors may act both as positive and negative regulators of the Wnt/β-catenin signaling dependent on the Wnt ligand presented. Their activity may also depend on the cellular context. The dual activity of these secreted proteins adds a new level of regulation to Wnt signaling in the extracellular space.

## Methods

### *Xenopus *embryo manipulations

*Xenopus *eggs were obtained from females injected with 300 IU of human chorionic gonadotrophin (Sigma), and were fertilized *in vitro*. Eggs were dejellied with 2% cysteine hydrochloride pH 8 and embryos were microinjected in 1XMBS-H (88 mM NaCl, 1 mM KCl, 2.4 mM NaHCO3, 0.82 mM MgSO4, 0.41 mM CaCl2, 0.33 mM Ca(NO3)2, 10 mM HEPES pH 7.4, 10 μg/ml streptomycin sulfate and 10 μg/ml penicillin). The embryos were cultured in 0.1XMBS-H and staged according to Nieuwkoop and Faber (1967) [31].

### Plasmid constructions and mRNA microinjections

Xfz4S cDNA was amplified from cDNA preparations of gastrula and neurula stages. The open reading frame of Xfz4S was amplified by PCR using following primers; 5'-ATGGGGGCAAGATCGCTGACCTTGTTGTAC-3' and 5'-CCTTGTGGTTTATAGGGAGAGGACACAGGC-3' and was cloned into pCS2+ plasmid. A part of the intron1 of *Xfz4 *(*Xfz4-intronI*, nt- 281-925 in Fig. 1A) used as an *in situ *probe to specifically detect the *Xfz4S *transcripts was amplified from the NF stage 19 cDNA preparation using the following primers: 5'-TTCACTCTACCAACGCGCAACTTACG-3' and 5'-GACACAGTCACTTTTTGTGGACGCTG-3' and was cloned into pCR-Blunt II-TOPO (Invitrogen). The ORF of Wnt-5a was amplified by PCR from pSP64T-Xwnt-5a [32] and was cloned into pCS2+ vector at *EcoRI *and *XhoI *sites. The extracellular amino terminus domain of human Frizzled 5 containing the first 233 amino acids was amplified by PCR using 5'-TTGCTGCTGCTCGGATCCGCCACCATGGCTC-3' and 5'-ATGGATCCCGTGCGCTCGTCGGCACTGAAG-3' primers and was cloned into pCS2+MT plasmid at *BamHI *site. Myc-tagged Fz4S and flag-tagged Wnt-5a were constructed by amplifying the respective ORFs by PCR and cloning them into pCS2+MT or pCS2+Flag plasmids (both gifts from Ralph Rupp). Both constructs contain the myc or flag tag at their C-terminus. All the constructs were verified by sequencing.

Capped mRNAs were synthesized from linearized plasmids using mMessage mMachine Kit (Ambion). *Wnt-3a *[33] (linearized with *EcoRI*, transcribed with SP6), *Wnt-4 *[34] and *Xdsh-DIX *[35] were linearized with *SalI *and transcribed with SP6. *Wnt-5a*, *NXfz8 *(*ECD8*) [36], *Xdkk1 *[37] and *Xdsh-dd2 *[38] and *Xfz4S *were linearized with *NotI *and transcribed with SP6. *Wnt-8b *[39], Δ*C-LRP6 *[9] and *NXfz7 *(*ECD7*) were linearized with *Asp718 *and transcribed with SP6. Synthetic mRNA from other constructs were prepared as follows: *Wnt-8 *(linearized with *BamHI *and transcribed with SP6) [40], *Wnt-11 *(linearized with *EcoRI*, transcribed with T7) [27], *Hfz5 *(linearized with *HindIII*, transcribed with SP6) [19] and *NHfz5 *(*ECD5*) was linearized with *BstXI *and transcribed with SP6.

### RT-PCR

Total RNA was prepared from embryos or animal cap explants with Trizol^® ^reagent (Invitrogen). First strand cDNA was synthesized with H minus M-MuLV reverse transcriptase (Fermentas) using random hexamers as primers. PCR was performed using standard conditions and the following sets of primers: Xfz4S-E1I1 (P1) '5-TTGTTGTACCTCCTGTGCTGCCTC-3' and '5-TGGTAGAGTGAAATGCGCAGCAGC-3' (271 bp, Tm 60°C and 29 cycles); Xfz4S-E2I2 (P2) '5-CATCAGGATCACCATGTGCCAG-3' and '5-GAAAGTAAACCCCCTGTGCTGAG-3' (277 bp, Tm 60°C, 29 cycles); *Xnr-3 *'5-TGAATCCACTTGTGCAGTTCC-3' and '5-GACAGTCTGTGTTACATGTCC-3' (233 bp, Tm 65°C, 29 cycles); ODC '5-GTCAATGATGGAGTGTATGGATC-3' and '5-TCCATTCCGCTCTCCTGAGCAC-3' (385 bp, Tm 65°C, 25 cycles).

### *In situ *hybridization

Whole mount *in situ *hybridization and antisense probe preparation was carried out as described [41]. Digoxigenin labelled antisense RNA was synthesized from plasmid containing *Xnr3 *(linearized with *EcoRI*), pCR-Blunt II-TOPO – *Xfz4-intronI *and pCR-Blunt II-TOPO – *Xfz4 *(both linearized with *BamHI*) using T7 RNA polymerase. Digoxigenin labelled sense RNA for *Xfz4-intronI *was synthesizes by linearizing the plasmid with NotI and transcribing with SP6.

### Co-immunoprecipitation

*Xenopus *embryos were injected with 500 pg myc-tagged Fz4S and 500 pg flag-tagged Wnt-5a mRNA at 2–4 cells stage. The embryos were grown until gastrula stage and protein was extracted in NP-40 lysis buffer (10 mM Tris-Hcl, pH 7.5, 100 mM NaCl, 2 mM EDTA, 1 mM EGTA, 0.5% NP-40, 5% glycerol with a cocktail of proteinase inhibitors). The embryo extract was incubated for 2 h either with 4 μg of anti-flag (M2, Sigma), 2 μg anti-myc (9E10, Calbiochem) or 2 μg of mouse IgG (Sigma) at 4°C with constant rotation. The samples were centrifuged and 30 μl of protein G beads (Pierce) was added to the supernatant. The beads were incubated with the protein extract for 2 h, centrifuged and washed four times with NP-40 lysis buffer. The immunoprecipitates were separated on 12% SDS-PAGE and were transferred to nitrocellulose membrane. For detection of immunoprecipitated proteins, the membranes were incubated with either anti-myc or anti-flag antibodies followed by incubation with peroxidase-conjugated secondary antibody. Bound secondary antibodies were visualized using SuperSignal west pico reagent (Pierce).

## Competing interests

The author(s) declare that they have no competing interests.

## Authors' contributions

RKS and HS designed the experiments. RKS performed most of the experiments and HS supervised the work. MK cloned the Xfz4 intron and established the genomic structure of Xfz4 gene. AM generated a new Xwnt-5a construct and performed *in situ *experiments. All authors contributed to writing and approved the final manuscript.
